# Relationships Between Achievement Goal Orientations, Learning Engagement, and Academic Adjustment in Freshmen: Variable-Centered and Person-Centered Approaches

**DOI:** 10.3389/fpsyg.2021.767886

**Published:** 2021-11-02

**Authors:** Haiying Wang, Mingxue Xu, Xiaochun Xie, Yuan Dong, Weichen Wang

**Affiliations:** School of Psychology, Northeast Normal University, Changchun, China

**Keywords:** freshmen, achievement goal orientations, learning engagement, academic adjustment, person-centered, variable-centered

## Abstract

Academic adjustment is a principal determining factor of undergraduate students’ academic achievement and success. However, studies pay little attention to freshmen’s antecedent variables of academic adjustment. This study aimed to examine the mechanisms underlying the relationship between achievement goal orientations and academic adjustment in freshmen using variable- and person-centered approaches. A sample of 578 freshmen (aged 18.29±1.04years, 58.5% female) completed questionnaires on achievement goal orientations, learning engagement, and academic adjustment. Latent profile analysis of achievement goal orientations revealed four groups: low-motivation (11.1%), approach-oriented (9.5%), average (52.8%), and multiple (26.6%). In the mediating analysis, results of the variable-centered approach showed that learning engagement mediated the effects of the mastery-approach and performance-avoidance goals on academic adjustment. For the person-centered approach, we selected the average type as the reference profile, and the analysis revealed that compared with the reference profile, learning engagement partially mediated the link between the approach-oriented profile and academic adjustment. The current study highlights the important role that achievement goal orientations and learning engagement play in academic adjustment. We discuss the implications and limitations of the findings.

## Introduction

When freshmen enter the campus, they encounter challenges in the form of a series of internal and external environmental changes, such as facing a new social environment, adjusting to new roles and responsibilities, and developing new academic and social relationships (Aderi et al., 2013). These challenges often contribute to various problems, such as anxiety, depression, loneliness, and withdrawal (Arjanggi and Kusumaningsih, 2016; Darlow et al., 2017; Lindell et al., 2020). Academic adjustment is a necessary component to overcome the above-mentioned challenges and plays a pivotal role in freshmen’s college adaptation. It refers to the degree of students’ adaptation to their academic demands, which include attitudes toward the curriculum, engagement with course material, and academic effort (Baker and Siryk, 1984).

To be competent in a new learning environment, students need to alter their customary learning habits and strategies (Awad et al., 2014). However, this is often accompanied by a lack of learning motivation and a decline in academic performance (Tuominen-Soini et al., 2012), which are further related to stress, anxiety, smoking, and alcohol consumption (Morrison and Cosden, 1997). To advance our understanding of academic adjustment and promote adjustment to campus life in freshmen, we explored the antecedents of academic adjustment.

Recently, investigators have demonstrated that numerous personal traits and social environments predict student academic adjustment. Factors include individual traits, parental relationships, social support, and motivations (Crede and Niehorster, 2012; van Rooij et al., 2018; Montgomery et al., 2019). Among these preconditions, achievement goal orientations are important (Kaplan and Maehr, 2007) because they are motivation-related constructs. They refer to the disposition reflecting the general tendency of students to select specific goals and favor specific outcomes in the context of achievement (Dweck, 1986). Several decades of research highlight the benefits of achievement goals in supporting students’ learning adjustment and engagement (Linnenbrink-Garcia and Wormington, 2019), whereby achievement goals help to influence adolescents’ maladaptive academic functioning and contribute to explaining and predicting behavior in an academic environment (Xu et al., 2020). Overall, mastery-approach and performance-approach goals indicate positive effects on the academic adjustment while mastery-avoidance and performance-avoidance goals indicate negative effects (Baranik et al., 2010; Van Yperen et al., 2015; Tian et al., 2017; Honicke et al., 2020). Most studies suggested that girls possess higher mastery-approach goals and lower performance-avoidance goals and boys are more performance-oriented than girls (Butler, 2014; Mouratidis et al., 2017; Yu and McLellan, 2019).

Previous studies primarily examined the direct effects of achievement goal orientations on academic adjustment. However, there is a lack of in-depth studies on how achievement goal orientations relate to academic adjustment. Based on the integrative development-in-sociocultural-context model, engagement is conceptualized as an enriching multidimensional construction shaped by interactions between the individual and the environment. The model emphasizes that engagement has been examined as a pathway or process through which personal factors shape learning outcomes (Skinner and Pitzer, 2012; Skinner et al., 2016). Firstly, students vary in their level of learning engagement. For the differences, the reason is their motivations in achievement goals (Daumiller et al., 2021). Bipp et al. (2021) pointed out that achievement goal orientations are important for students to ensure quality learning experiences in the school environment. Secondly, learning engagement plays a prominent role in shaping adolescents’ adjustment and is an important construct to promote learning among college students (Poortvliet et al., 2015; Skinner et al., 2016). Engagement, as outlined by Schaufeli et al. (2002), is a better construct to understand what makes students go the extra mile, and it purports to measure students’ psychological presence and involvement in their study. Also, engagement means an emerging learning attitude. Therefore, based on these findings, we investigated whether achievement goal orientations affect academic adjustment *via* learning engagement.

To date, most studies in the achievement goal orientations field have focused only on the variable-centered approach which expressed how different forms of specific goals were uniquely and independently related to specific outcomes (Linnenbrink-Garcia and Wormington, 2019). However, this approach ignores potential variability and differences among individuals, and can mask vital individual patterns of achievement goal orientations within subgroups of individuals (Tuominen-Soini et al., 2012). For example, it is obviously difficult to distinguish the students who only take mastery goals and students who take both mastery goals and performance goals. In contrast, the multiple goals perspective of the person-centered approach classifies students into homogenous groups with similar goal orientation profiles and is benefit to learn the students’ generalized motivational tendencies (see Niemivirta, 2002). The person-centered approach of achievement goal orientations suggests that students may pursue multiple goals simultaneously or seek to attain a single outcome for multiple reasons or serve multiple functions by striving for certain goals in the school environment (Tuominen-Soini et al., 2008, 2011). Wormington and Linnenbrink-Garcia (2017) indicated that the person-centered approach, which provides an accurate representation of multiple goal pursuits and broadens the consideration of multiple goals, can extend the findings obtained using the variable-centered approach. In fact, the variable-centered and person-centered approach are closely related and complement each other (Bergman and Trost, 2006), and the combination comprehensively discussed the association between achievement goal orientations, learning engagement, and academic adjustment. Neither of these single approaches is sufficient to study achievement goal orientations accurately. Researchers can collectively benefit from the results of variable-centered and person-centered analyses and acquire valuable information on achievement goal theory. It is worthwhile to mention that few studies have used a person-centered approach, and even fewer have applied a combination of these two approaches. Therefore, in this study, we extended this prior work by focusing on a broader set of achievement goal orientations, aiming to investigate the interactions between achievement goal orientations, learning engagement, and academic adjustment by combining the two techniques.

### Investigation of the Profiles of Achievement Goal Orientations

Person-centered approach in achievement goals represents multiple goal pursuit which complement variable-centered findings and expand the consideration of multiple goals beyond mastery-approach and performance-approach goals alone. The approach is meaningful to provide a clearer understanding of which combinations of goals typically emerge in a population. There has been extensive work on classifying achievement goal orientations, which comprise five main combinations: a basic mastery profile [i.e., mastery- and learning-oriented (Tapola and Niemivirta, 2008; Tapola et al., 2014; Peixoto et al., 2016)], a basic performance profile [i.e., performance-oriented and low-mastery/high-performance (Tuominen-Soini et al., 2012; Gonçalves et al., 2017)], a mastery and performance-approach goals combined profile [i.e., success-oriented, multiple goals cluster, and approach-oriented group (Daniels et al., 2008; Tuominen-Soini et al., 2008, 2011; Luo et al., 2011; Zhang et al., 2016)], a moderate or low profile [i.e., indifferent, average goals (Tuominen-Soini et al., 2008, 2011; Jansen In de Wal et al., 2016) low-mastery/low-performance, low-motivation, and disaffected (Daniels et al., 2008; Gonçalves et al., 2017)], and a profile emphasizing avoidance [i.e., avoidance-oriented students (Daniels et al., 2008; Tuominen-Soini et al., 2008, 2012)]. In this study, we employed the latent profile analysis (LPA), a person-centered approach, to classify the research objects. LPA is useful in describing the types of achievement goal orientations and is optimally suited to identify which individuals may benefit from different combination of goals to adapt the learning environment. Therefore, the present study aimed to do the similar classification in Chinese freshmen. We expected to detect several meaningful types of achievement goal orientations, which highlighted either a single or a combination of goal orientations. Based on the previous literature, we hypothesized that we would identify groups of students with five dominant tendencies of mastery-oriented, performance-oriented, approach-oriented, avoidance-oriented, and low-motivation (Hypothesis 1). We identified common patterns of goals profiles first and then compared the benefits of endorsing different goal profiles with learning engagement and academic adjustment.

### Achievement Goal Orientations and Academic Adjustment

The achievement goal theory is one of the most influential theories to explain and predict the direction and intensity of individuals’ behavior in school-related situations (Xiang et al., 2017), which has experienced a 30year change in processes. Initially, achievement goal orientations were characterized by students’ aims for task engagement, which were differentiated into two distinct types: mastery goals that focused on the development of competence and performance goals that were concerned with the demonstration of competence (Dweck, 1986). Subsequently, Elliot and Harackiewicz (1996) refined achievement goal orientations and established two types of performance goals. Elliot and McGregor (2001) proposed a more detailed extension that further divided mastery goals into approach and avoidance components, and a 2×2 model of achievement goal orientations was developed and tested. The quartered model emphasized two directions: the definition of competence and the valence of competence. The former refers to whether the individual considers their ability selectable (mastery and performance goals), whereas the latter refers to whether an individual pays attention to achieving positive outcomes (i.e., success; approach-oriented) or avoiding negative outcomes (i.e., failure; avoid-oriented). Specifically, mastery-approach goals are related to the motivation for improving knowledge, skills, and learning, whereas mastery-avoidance goals are concerned with the purpose of avoiding misunderstandings or failing to accomplish a task. Performance-approach goals focus on the endeavors to outperform others and demonstrate their superiority, whereas performance-avoidance goals represent the desire to avoid performing more poorly than others.

Recently, from the perspective of the variable-centered approach, investigators confirmed the effectiveness of the dimensions of achievement goal orientations on academic adjustment (Linnenbrink-Garcia and Wormington, 2019). These studies established that the direct relationships between the mastery-approach and performance-avoidance goals on academic adjustment are relatively consistent; however, some uncertainty remains regarding the relationship between the mastery-avoidance and performance-approach goals and academic adjustment. Specifically, mastery-approach goals are positively associated with adaptive coping strategies and positive behavior patterns, such as high levels of effort, persistence at a task, and adoption of deep learning strategies (Honicke et al., 2020). Therefore, we proposed Hypothesis 2a: Mastery-approach goals will positively predict academic adjustment.

Performance-avoidance goals are mainly associated with unfavorable results and maladaptive adjustment patterns (Van Yperen et al., 2014, 2015). Students who possessed performance-avoidance goals have a tendency to avoid learning, choose disorganized study strategies, or fail to use learning strategies. They exhibit high rates of burnout, lower self-efficacy, inactive learning interest, anxiety, shame, and hopelessness (Pekrun et al., 2006; Auvinen et al., 2015; Nadon et al., 2020). Thus, we proposed Hypothesis 2b: Performance-avoidance goals negatively predict academic adjustment.

Several studies have revealed that mastery-avoidance goals are linked to adaptive outcomes, such as learning interest (Baranik et al., 2010), whereas other studies have suggested that mastery-avoidance goals are more likely to be related to maladaptive outcomes, such as procrastination, disorganization, and declines in performance (Howell and Watson, 2007; Van Yperen et al., 2009). Thus, we proposed Hypothesis 2c: Mastery-avoidance goals negatively predict academic adjustment.

There is another ambiguous relationship between performance-approach goals and academic adjustment. Meece et al. (2006) showed that performance-approach goals are positively correlated with self-handicapping strategies and lower academic help-seeking. However, other researchers have found that these goals were positively correlated with adaptive behavior patterns, such as using learning strategies and making effort (Mouratidis et al., 2013; Tian et al., 2017). In collectivist cultural contexts, performance-approach goals are more likely to be associated with positive learning outcomes (Cheng and Lam, 2013; Datu, 2018). Therefore, taken together, we proposed Hypothesis 2d: Performance-approach goals positively predict academic adjustment.

From the person-centered approach perspective and based on LPA results, researchers have explored the relationships between the profiles of achievement goal orientations and academic adjustment and found that the combination of goals predicting relevant adaptive outcomes of education remains controversial (Tuominen-Soini et al., 2012). Firstly, students with high mastery-oriented goals showed stronger ability to adapt to consequences and showed more adaptive patterns of motivation and academic wellbeing than those who possessed weak mastery ability. At the same time, mastery-oriented students may engage more time studying (Tapola and Niemivirta, 2008; Tuominen-Soini et al., 2012). Secondly, students who focused on both mastery- and performance-approach goals (approach-oriented goas) had two-sided adaptive outcomes (Daniels et al., 2008; Tuominen-Soini et al., 2008, 2011, 2012; Luo et al., 2011). On the one hand, several studies have suggested that these students are more likely to use cognitive strategies and make effort to achieve better academic performance and encounter difficulties than students with low motivation. Also, approach-oriented students are higher on self-efficacy, time management, and meta-cognitive self-regulation than are mastery-oriented students. On the other hand, other studies reported that these students experienced several positive outcomes, such as lower levels of anxiety and negative affect. Thirdly, because of the stronger concerns with performance, students with performance-oriented goals showed more school burnout, higher test anxiety, and higher negative affect than students in approach-oriented and mastery-oriented goals groups (Luo et al., 2011; Tuominen-Soini et al., 2012). Finally, students with low-mastery performance [low-motivation cluster (Daniels et al., 2008) and indifferent students (Tuominen-Soini et al., 2008, 2011)] or avoidance-oriented goals (Daniels et al., 2008; Tuominen-Soini et al., 2008, 2012) were similarly to those who were low in motivation and academic adjustment. Given the above-mentioned evidence, we hypothesized that from high to low level of goals’ scores on adaptive academic achievement, these types are approach-oriented, mastery-oriented, and performance-oriented goals. The avoidance-oriented and low-motivation goals negatively predict academic adjustment (Hypothesis 3). However, the mechanisms underlying the relationship between achievement goal orientations and academic adjustment remain to be elucidated. It is important to gain insight into how achievement goal orientations affect academic adjustment. Therefore, this study sought to provide guidance for educational practitioners by exploring the effects of the mediating variable of learning engagement.

### The Mediation of Learning Engagement

The integrative development-in-sociocultural-context model elaborates on the function of engagement in the key facilitators and consequences of identity engagement (Wang et al., 2019). The model emphasizes that engagement plays a dynamic developmental and reciprocal role in shaping youth’s learning processes. In this model, engagement is conceptualized as an enriching multidimensional construction shaped by interactions between the individual and the environment. On the one hand, personal beliefs (e.g., competence beliefs) have been established as antecedents of engagement (Wang and Eccles, 2013). On the other hand, longitudinal studies have shown that engagement predicts children’s educational outcomes, such as academic achievement (Li et al., 2019).

Learning engagement is a multifaceted construct in nature and mainly reflects individuals’ varying patterns in cognition, emotion, and behavior (Phan, 2016). Schaufeli et al. (2002) were interested in the multifaceted composition of learning engagement and defined it as a persistent, positive, fulfilling, and learning-related state of mind that consists of three major motivation-related components: vigor, dedication, and absorption. Vigor refers to a high level of energy, resilience, willingness, and ability to study, and persistence in the face of difficulties. Dedication refers to a strong involvement in studying, accompanied by a sense of significance, enthusiasm, pride, and inspiration. Absorption refers to a pleasant state of complete immersion in studying, which is characterized by being unable to detach oneself from studying. Learning engagement is defined and highlighted by Schaufeli et al. (2002) as focusing on the positive aspects of a person’s study and having educational implications. Studies have found that learning engagement is a principal determining factor of students’ adjustment and achievement in educational settings (García-Ros et al., 2018; León et al., 2021; León and García-Martínez, 2021). Therefore, we would more fully explore the learning engagement and how learning engagement interacts with other education-related factors.

Studies using the variable-centered approach have demonstrated that learning engagement contributes to academic adjustment. Students who are highly engaged in work follow an overall positive adolescent development trajectory (Upadyaya and Salmela-Aro, 2013). Learning engagement is correlated with several academic aspects, such as adaptive coping strategies (e.g., meta-cognitive strategies), self-efficacy, performance, and academic success, and it is negatively correlated to test anxiety and ill-being (Upadyaya and Salmela-Aro, 2013; García-Ros et al., 2018; Vizoso et al., 2018). High learning engagement also improves students’ wellbeing, which includes positive affect and life satisfaction (Upadyaya and Salmela-Aro, 2013).

According to the development-in-sociocultural-context model, developmental competencies and motivational beliefs work together to shape engagement (Wang et al., 2019). Achievement goal orientations as motivation-related constructs (Wigfield and Cambria, 2010) are predictors of adolescents’ learning engagement (Shih, 2012), and important, albeit limited, empirical evidence has established such links. Specifically, both mastery-approach goals (Shih, 2012; Poortvliet and Perdeck, 2014; Nerstad et al., 2020; van Dam et al., 2020) and performance-approach goals (Shih, 2012; Barashev and Li, 2017) are positively related to learning engagement. Mastery-avoidance goals are unrelated to learning engagement (Poortvliet and Perdeck, 2014); however, studies have frequently found that they are positive predictors of work exhaustion (de Lange et al., 2010; Poortvliet et al., 2015). Furthermore, performance-avoidance goals negatively predict learning engagement (Shih, 2012; Barashev and Li, 2017).

For the person-centered approach, Wormington and Linnenbrink-Garcia (2017) developed person-centered approaches to study achievement goals using meta-analytical techniques. They found that mastery- and approach-oriented profiles are adaptive profiles, and students with mastery-oriented goals had higher engagement than those with approach-oriented goals. Average and low all-goal profiles have shown the lowest levels of engagement. Moreover, the effects of high-performance approach and performance-avoidance profiles on engagement did not differ. However, Liu et al. (2020) reported that in Chinese educational contexts, the approach-oriented profile is the most adaptive to learning engagement, followed by the mastery-oriented profile.

In summary, from the perspective of variables, we proposed the following hypotheses: Mastery-approach and performance-approach goals positively and independently predict academic adjustment through learning engagement (Hypotheses 4a and b); mastery-avoidance and performance-avoidance goals negatively and independently predict academic adjustment through learning engagement (Hypotheses 4c and d). At the individual level, we predicted that approach-oriented (Hypothesis 5a) and mastery-oriented goals (Hypothesis 5b) would positively influence academic adjustment through learning engagement.

## The Current Study

The variable-centered approach is helpful to quantify the individual variables’ effects while controlling for potential confounds (Warren et al., 2021), but this approach might conceal important results and implications (Stern and Hertel, 2020). The person-centered approach instead examines how the combination of variables to function together and mainly focuses on subgroups of individuals with the most common patterns of scores observed according to the data (Lanza and Cooper, 2016). However, no previous study combined the both approach in achievement goal orientations field. In this study, we integrated information gathered using the person-centered approach with that gathered using the mainstream variable-centered approach and explored the goal pursuits of students. We identified the dimensions and profiles of achievement goal orientations that influenced academic adjustment and investigated whether learning engagement mediates the relationship between achievement goal orientations and academic adjustment. Firstly, we used LPA to study the profiles of achievement goal orientations in freshmen. Then, building on the variable-centered approach, we analyzed the mediating role of learning engagement on dimensions of achievement goal orientations and academic adjustment. Finally, based on the results of the LPA, we further explored whether learning engagement plays a mediating role in potential profiles of achievement goal orientations and academic adjustment. Taken together, we assessed the direct effect of achievement goal orientations on academic adjustment and the underlying mechanisms of this effect.

## Materials and Methods

### Participants and Procedures

We estimated sample size by G*Power and calculated the minimum sample size. Based on our preliminary research, we set an alpha of 0.05, power (1−*β*) of 0.80, and an effect size of 0.04. The sample size was 191. In this study, a total of 590 college students were recruited from universities in northeast China using random sampling. Due to missing or invalid responses, 12 participants were not included in the analyses. Therefore, the final sample consisted of 578 participants (338 females, 58.5%). Mean age was 18.29 years [standard deviation (SD)=1.04]. During the first month of the new semester (in October 2018), the first author with the assistance of teachers used questionnaires to assess freshmen’s goals, learning engagement, and academic adjustment at a public university located in China. Participants were informed that all information would be kept confidential and that they could withdraw from the study at any time. Questionnaires took 5min to complete. The study was approved by the Academic Ethics Committee of the College of Psychology of Northeast Normal University.

### Measures

#### Achievement Goal Orientations

An adaptation of the 29-item achievement goal orientations questionnaire (Liu and Guo, 2003) was used to measure the four dimensions of achievement goal orientations: mastery-approach (nine items, e.g., “In class, my goal is to learn as much as possible”), mastery-avoidance (five items, e.g., “When I study, the most worries are that others think of me as stupid”), performance-approach (nine items, e.g., “In class, my goal is to be better than others”), and performance-avoidance (six items, e.g., “I won’t ask the teacher questions that I can’t solve by myself, because I don’t want the teacher to think that I’m stupid”). Participants rated each item on a five-point scale from 1=not true to 5=certainly true. In this study, the reliability of the sub-scales in this study was in order 0.74, 0.71, 0.82, and 0.81, and the total alpha coefficient was 0.87.

#### Learning Engagement

We used the learning engagement subscale from the Utrecht Work Engagement Scale-Student (UWES-S; Schaufeli et al., 2002), revised by Fang et al. (2008). It measures learning engagement on along dimensions: vigor, dedication, and absorption. The final version of the scale consisted of 17 items (e.g., “At my academic work, I feel as though I am bursting with energy”) and all items were rated on a seven-point Likert scale (1=Never to 7=Always), where a higher score indicated a higher level of learning engagement. In this study, the reliability of the sub-scales in this study was in order 0.86, 0.88, and 0.88, and the total alpha coefficient was 0.94.

#### Academic Adjustment

Academic adjustment was measured using the 29-item academic adjustment of undergraduates in China scale (Feng et al., 2006). It comprised five factors: learning motivation, learning ability, teaching model, learning attitude, and learning environment (e.g., “I can’t adapt to the college schedule”). On a five-point Likert scale (1=not true to 5=certainly true), students were asked to rate how strongly they agreed with each statement. In this study, the reliability of the sub-scales in this study was in order 0.75, 0.77, 0.78, 0.72, and 0.62, and the total alpha coefficient was 0.84.

### Statistical Analyses

Mardia’s Skewness and Mardia’s Kurtosis were used to assess multivariate distribution by the online WebPower (Liu, 2013). Results showed Skewness (*b*=2.75, *z*=264.68, *p*<0.05) and Kurtosis (*b*=57.23, *z*=11.33, *p*<0.05), and indicated the absence of multivariate normality. Knief and Forstmeier (2018) suggested that comparing with other violations or alternative approaches, violations of multivariate normality may be less detrimental to the interpretation of findings, so we choose to process with the analyses using 1,000 bootstraping (Pek et al., 2018). To test our hypotheses, we performed five steps to conduct the variable-centered and person-centered analyses and controlled for sex. Preliminary analyses included descriptive statistics and correlation analysis. Second, we used LPA to identify profiles along the dimensions of achievement goal orientations using Mplus8.0 (Muthén and Muthén, 1998–2015) and validated the efficacy of the classifiers. Third, we performed ANOVAs and *post-hoc* comparisons (Bonferroni adjustment) to analyze the relationships between achievement goal orientations types, learning engagement, and academic adjustment. Fourth, we performed a mediation analysis among the three variables using MEDIATE in SPSS 22.0 (Hayes and Scharkow, 2013). Finally, we tested the mediation effect of learning engagement on achievement goal orientations profiles and academic adjustment using the bootstrap method by running the PROCESS plugin in the SPSS 22.0 software (Hayes, 2018). In the PROCESS, we used the ordinary least squares (OLS) to estimate the parameter in mediation model.

To explore the potential profiles of freshmen’s achievement goal orientations, potential profiles were analyzed by fitting a model that varied in solutions from 2 to 5 classes. Following Nylund’s (2007) suggestion, model fit comparisons are based on the following indicators: Akaike’s information criterion (AIC), Bayesian information criterion (BIC), adjusted BIC (aBIC), Lo-Mendell-Rubin likelihood ratio (LMR-LRT), bootstrap likelihood ratio (BLRT), and entropy. The model with a smaller AIC, BIC, and ABIC is better. If values of *p* of the LMR-LRT and BLRT are less than 0.05, this implies that the k-class model is significantly better than the k-1-class model. Higher entropy indicates few classification errors.

We investigated subsequent mediation effects by comparing the effects of profiles and learning engagement on academic adjustment. Based on the LPA, we explored the mediating role of learning engagement in achievement goal orientations profiles and academic adjustment (hypotheses 3 and 5). When the independent variable was multi-categorical, we used the method that integrated relative and omnibus mediation to analyze the mediation effect (Iacobucci, 2012). The first step was to implement an omnibus mediation analysis. Following previous methods, if the omnibus mediation effect is not significantly different from zero, the k-1 relative mediation effect is zero, where k reflects the number of profiles. Otherwise, the second step is applied, which involves conducting a relative mediation analysis that is aimed at determining the relative mediation effect. If the effect is significantly different from zero, then, the third step should be implemented. Otherwise, the mediation analysis is complete. In the third step, the relative direct effects are reported. Combining the characteristics and analysis results of achievement goal orientations profiles, we selected the average profile as the reference profile that was considered maladaptive (Tuominen-Soini et al., 2008), and the remaining profiles were compared with profile 3.

## Results

### Preliminary Analyses

A previous study found that girls were superior to boys in achievement goal orientations, which indicated that girls possessed higher mastery-approach goals and lower performance-avoidance goals (Mouratidis et al., 2017). Male students are more performance-oriented than females (Butler, 2014; Yu and McLellan, 2019). Moreover, girls were more apt at engaging in learning than are boys (Li et al., 2011; Salmela-Aro and Upadaya, 2012; Wang and Eccles, 2012). From this, we controlled for sex for all data analyses. The relationships between all variables were analyzed using partial correlation analysis. Means, SDs, and zero-order correlations among variables are shown in Table 1. As shown in Table 1, mastery-avoidance goals did not correlate with learning engagement or academic adjustment; therefore, we excluded it from the mediation analysis of the variable-centered approach.

**Table 1:** Means, standard deviations, and correlations (*n*=578) of the core variables.

S. No.		1	2	3	4	5	6
1.	Mastery-approach goals	–					
2.	Mastery-avoidance goals	0.30^***^	–				
3.	Performance-approach goals	0.46^***^	0.38^***^	–			
4.	Performance-avoidance goals	0.02	0.33^***^	0.31^***^	–		
5.	Learning engagement	0.47^***^	0.03	0.17^***^	−0.12^**^	–	
6.	Academic adjustment	0.42^***^	−0.1	0.10^*^	−0.33^***^	0.43^***^	–
	M	3.51	3.65	3.48	2.69	4.25	3.38
	SD	0.58	0.71	0.69	0.87	1.07	0.46

* *p<0.05,*

** *p<0.01, and*

*** *p<0.001*.

### Latent Profile Analyses

Researchers classify the latent profiles and understand the proportion of people of the various categories throughout the group according to the answer mode on the individual external test topic, rather than determine the number of classifications *a priori*. LPA is particularly suitable for exploratory research questions and offers several advantages (Stern and Hertel, 2020). This probabilistic model-based classification method can not only guarantee the largest difference between the divided categories and the smallest difference within the categories but also can be measured by objective statistical indicators. As shown in Table 2, the AIC, BIC, aBIC, entropy, and LMR-LRT results for the different classes indicated that the five-class solution did not fit the data better than did the four-class solution; thus, we chose the four-class solution.

**Table 2:** Model fit indices of the achievement goal orientations.

Model	AIC	BIC	aBIC	Entropy	LMR-LRT (*p*)	BLRT (*p*)
2-Class	6338.35	6395.02	6353.75	0.62	<0.001	<0.001
3-Class	6272.66	6351.13	6293.99	0.72	<0.01	<0.001
4-Class	6190.54	6290.81	6217.80	0.75	<0.01	<0.001
5-Class	6170.58	6292.65	6203.76	0.80	0.10	<0.001

**Figure 1:**
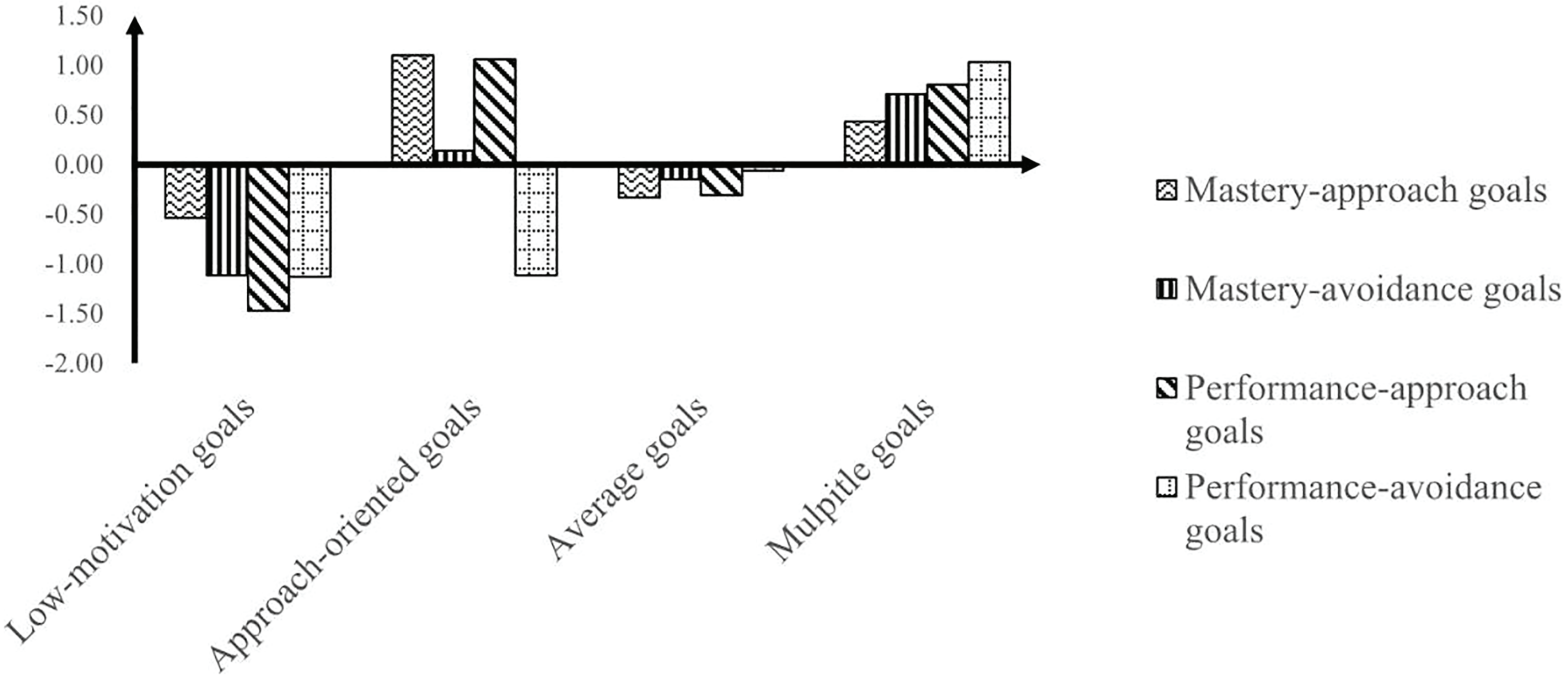
The standardized mean scores on achievement goal orientations across four profiles.

Figure 1 shows that the values for each variable were standardized scores per profile. Following the profile division method of achievement goal orientations used by Luo et al. (2011), we used a standardized score of 0.50 to divide and name the goals. We defined three levels: high (>0.50 SDs), average level (0.50–0.50 SDs), and low (<0.50 SDs). Profile 1 (11.61%) was characterized by low levels across all indicators of achievement goal orientations. We defined this class as low-motivation goals. Profile 2 (10.38%) included goals where scores of the two approach goals were both more than twice the 0.50 SD (the average level); the scores of the mastery-avoidance goals were contained within the average level, and performance-avoidance scores were sufficiently below the low level. We defined this class as approach-oriented goals. Profile 3 was the most prevalent (50.90%) and showed that the dimension scores of achievement goal orientations all fell within the average level. We defined this class as average goals. Profile 4 was the opposite of profile 1 and described 27.12% of the sample. This profile showed that mastery-approach goal scores were slightly below the high level, and the scores of the other three dimensions of achievement goal orientations were above the high level. We defined this class as multiple goals. Overall, LPA of achievement goal orientations revealed four groups: low-motivation (profile 1, 11.1%), approach-oriented (profile 2, 9.5%), average (profile 3, 52.8%), and multiple (profile 4, 26.6%).

To assess the validity of the classification of the profiles, we examined the association with the dimensions of achievement goal orientations using analyses of covariance. Table 3 shows the means and SDs of each goal orientation.

**Table 3:** Descriptive statistics (means±standard deviations), MANOWA, and *post-hoc* analyses of the relationships between latent profile analysis membership, achievement goal orientations, learning engagement, and academic adjustment.

Profile	*N*	mastery-approach goals	mastery-avoidance goals	performance-approach goals	performance-avoidance goals	Learning engagement	Academic adjustment
Profile 1	64	3.20±0.55	2.77±0.68	2.40±0.49	1.63±0.48	4.14±1.13	3.46±0.45
Profile 2	55	4.20±0.46	3.74±0.77	4.28±0.38	1.66±0.48	4.98±1.19	3.85±0.50
Profile 3	305	3.32±0.46	3.53±0.57	3.26±0.42	2.62±0.57	4.19±0.86	3.34±0.40
Profile 4	154	3.78±0.52	4.20±0.47	4.07±0.39	3.63±0.51	4.17±1.28	3.26±0.46
*F*(3,574)		75.83^***^	99.04^***^	347.01^***^	311.38^***^	9.74^***^	27.26^***^
Bonferroni		1=3<4<2	1<3=2<4	1<3<4<2	1=2<3<4	1=3=4<2	3=4<1<2
Effects (*η*^2^)		0.28	0.34	0.64	0.62	0.05	0.13

*** *p<0.001*.

### Relationships Between Achievement Goal Orientations Profiles, Learning Engagement, and Academic Adjustment

Results showed that there were significant differences in learning engagement [*F*(3,574)=9.74, *p*<0.001, *η*^2^=0.05] and academic adjustment [*F*(3,574)=27.26, *p*<0.001, *η*^2^=0.13] between the achievement goal orientations profiles. As shown in Table 3, *post-hoc* Bonferroni tests found that profile 2 (approach-oriented goals) had the highest scores for learning engagement and academic adjustment, and the academic adjustment score of profile 1 was significantly higher than that of profiles 3 and 4. According to the results of ANOVAs and *post-hoc* comparisons analyses, approach-oriented approach had the highest score for academic adjustment, low-motivation goals had the second highest score, and the rest of two goals had the lowest scores that were not significant. In the score of learning engagement, approach-oriented goals had the highest score, the rest three goals had low scores and revealed no significant differences.

### Mediation Analyses: Variable-Centered Approach

**Figure 2:**
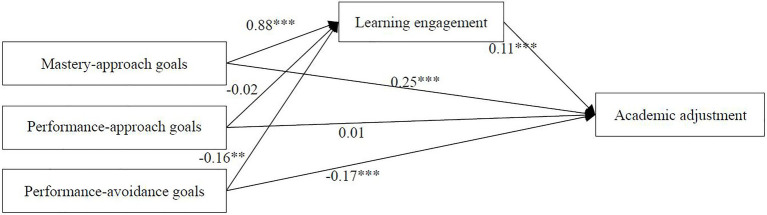
The proposed mediated model on variable level. ^**^p<0.01, ^***^p<0.001.

Because of the continuous variables, we used a simple mediation handler to test hypotheses 2 and 4 using the MEDIATE macro (Hayes and Scharkow, 2013). Figure 2 shows that mastery-approach goals were positively correlated with learning engagement (*β*=0.88, *p*<0.001) and academic adjustment (*β*=0.34, *p*<0.001), performance-avoidance goals were negatively correlated with learning engagement (*β*=−0.16, *p*=0.001) and academic adjustment (*β*=−0.19, *p*<0.001), and performance-approach goals were not significantly correlated with learning engagement (*β*=−0.02, *p*=0.81) or academic adjustment (*β*=0.01, *p*=0.80). In addition, learning engagement was positively correlated with academic adjustment (*β*=0.11, *p*<0.001).

Further statistical tests revealed that learning engagement mediated the relationship between mastery-approach goals and academic adjustment [indirect effect=0.09, 95% confidence interval (CI)=(0.06, 0.14)]. The mediation effect accounted for 26.47% of the total effect. Learning engagement mediated the relationship between performance-avoidance goals and academic adjustment [indirect effect=−0.02, 95% CI=(−0.03, −0.01)]. The mediation effect accounted for 11.11% of the total effect. From these results, we can make the conclusion that learning engagement mediated the effects of the mastery-approach and performance-avoidance goals on academic adjustment.

### Mediation Analyses: Person-Centered Approach

Because of the classified independent variable, the PROCESS macro in SPSS22.0 was used to analyze the mediation effect (Hayes, 2018). The omnibus mediation effects [*F*(3, 573)=27.08, *p*<0.001] and the relative mediation effect [*F*(3, 572)=19.72, *p*<0.001] were significantly different from zero.

**Figure 3:**
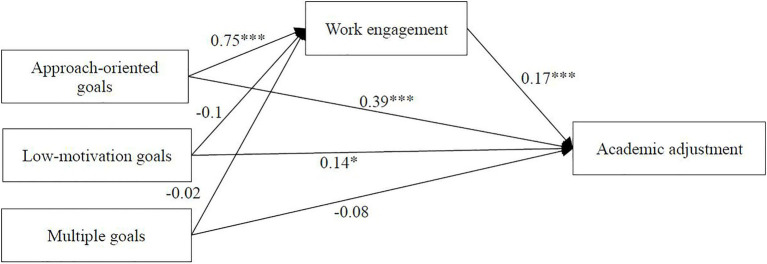
The proposed mediated model on individual level. ^*^p<0.05, ^***^p<0.001.

Results of the relative mediation analysis showed that low-motivation goals directly predicted academic adjustment. However, the mediation results were not significant because the CIs included zero. Therefore, they are not reported. However, the 95% bootstrap CIs (0.06, 0.19) of the relative mediation of approach-oriented goals excluded 0, which indicated that the relative mediation effect was significant (a_1_=0.75, b=0.17, a_1_b=0.13). That is, freshmen with approach-oriented goals had 0.75 times greater learning engagement level than those with approach-oriented goals (a_1_=0.75), and their academic adjustment level also increased by 0.17 (b=0.17) with learning engagement (Figure 3). The relative direct effect of approach-oriented goals was significant (c'_1_=0.39, *p*<0.001), which indicated that academic adjustment in students with approach-oriented goals was 0.39 higher than that of students with average goals. Moreover, the relative total effect was significant (c_1_=0.51, *p*<0.001), and the relative mediation effect a_1_b was 25.49%. From these results, we can make the conclusion that learning engagement played a mediated role between approach-oriented goals and academic adjustment.

## Discussion

We investigated the relationships between achievement goal orientations and freshmen academic adjustment as well as the mediating role of learning engagement by combining variable- and person-centered analyses. Results revealed four potential profiles of achievement goal orientations. Learning engagement played a mediating role between two dimensions of achievement goal orientations (mastery-approach and performance-avoidance goals) and academic adjustment. The approach-oriented profile positively correlated with academic adjustment *via* learning engagement.

### Achievement Goal Orientations Profiles

We explored the latent profiles of achievement goal orientations by conducting an LPA. Inconsistent with hypothesis 1, the LPA revealed four profiles of achievement goal orientations in freshmen: low-motivation, approach-oriented, average, and multiple. This inconsistency may be due to unknown factors that resulted in individual variations (Lo et al., 2017). The average profile had the highest number of freshmen (50.9%), and the approach-oriented students had the lowest number (10.4%). These results can be deceiving for the following three reasons. First, the cultural background of Chinese Confucian-collectivism emphasizes harmony and balance over individual achievement and is focused on the interconnectedness of everyone and everything (Gardiner et al., 2020). Second, students’ hard work and mastery of knowledge are aimed at showcasing their talents or gaining recognition from external factors (e.g., parental expectations), which are likely to result in a lack of initiative. External factors may further increase students’ fear of academic failure, which may cause them to refuse to pursue specific goals (Lin et al., 2003; Shih, 2008). Third, holding multiple goals is common in school because students may seek to follow personal interests and respond to external demands (Tuominen et al., 2020).

In addition, we revealed other achievement goal orientations profiles. Multiple profile students (27.1%) focused more on achieving better grades than other students and avoiding failure and mistakes. This profile emphasized not only learning and mastering knowledge in school but also achieving good grades and performing better than others. However, low-motivation profile students (11.6%) had relatively low scores across all dimensions of achievement goal orientations and did not focus on specific goals, such as mastering knowledge, performing better than others, or avoiding failure. Overall, these results indicated that different dimensions of achievement goal orientations can coexist in a student, and the variable-centered approach for exploring achievement goal orientations may lead to inappropriate conclusions (Ryan and Shim, 2006, 2008).

### Relationship Between Achievement Goal Orientations and Academic Adjustment

In the current study, we tested the relationships between achievement goal orientations and academic adjustment, and the results partially supported our hypotheses. Specifically, mastery-approach goals positively predicted academic adjustment (hypothesis 2a), and performance-avoidance goals showed the opposite pattern (hypothesis 2b). These results support Dweck’s (1986) point that an individual who has mastery-approach goals develops adaptive behavior, whereas one who has performance-avoidance goals mainly develops maladaptive behavior (Gillet et al., 2015).

However, performance-approach goals were negatively correlated with academic adjustment, which was contrary to hypothesis 2d. A possible explanation is that goal realization is brought about contrary results by the different self-determination reasons behind goals (Elliot and Murayama, 2008). For example, the autonomous reasons underlying performance-approach goals are closely related to the adaptive results (e.g., persistence), whereas the control reasons underlying performance-approach goals are closely associated with maladaptive results (e.g., negative affect; Vansteenkiste et al., 2010). In the future, distinguishing achievement goals from achievement goal orientations and examining the underlying reasons for adopting and pursuing achievement goals is warranted.

Mastery-avoidance goals were shown to be unrelated to academic adjustment, which rejects hypothesis 2c. This may be because mastery-avoidance goals have both positive (emphasizing learning and striving toward goals that imply self-improvement and growth) and negative (avoiding failing) characteristics (Michou et al., 2016). The interaction between mastery-avoidance goals and outcomes depends on the characteristics of the individual that are more prominent (Elliot, 1999); therefore, the results are inconsistent. Another possible reason is that compared with freshmen, higher-grade students are more likely to experience or perceive some loss of skills or abilities with increasing grade due to the increasing amount of course content and difficulty. Therefore, it can be predicted academic adjustment more accurately in older students (de Lange et al., 2010; Senko and Freund, 2015).

From the person-centered approach, we found that students with approach-oriented goals had the highest scores for academic adjustment, and goals directly predicted academic adjustment. Results were concordant with hypothesis 3a and consistent with previous research by Tuominen-Soini et al. (2008). In addition, low-motivation goals directly predicted academic adjustment, whereas multiple goals did not. Notably, if scores of the approach-oriented goals are largely in line with (see low-motivation) or below (see multiple goals) scores of the avoidance-oriented goals, motivation-oriented goals may or may not positively predict academic adjustment. However, when approach-oriented goal total scores are higher than those of avoidance-oriented goals, it may predict higher academic adjustment (see approach-oriented goals). As mentioned in the Introduction, achievement goal orientations refer to the channels through which achievement-related motive dispositions manifest in students during specific achievement situations, and in the hierarchical model of achievement motivation (Elliot and Church, 1997; Elliot, 1999), motive dispositions reflect competence- and affect-based constructs that promote individuals to adopt achievement goals and orient them toward success (approach-oriented) or away from failure (avoidance-oriented) in specific achievement contexts and situations. Approach-oriented goals are focused on the constructive aspects of achievement, whereas avoidance-oriented goals are focused on the negative aspects (Job et al., 2009). Therefore, we can infer that regardless of the avoidance-oriented goals that the individual adopts, as long as it is accompanied by similar or a higher degree of approach-oriented goals, both motivation-oriented goals have a positive impact on the adaptation of the individual. Another notable finding is that individuals with mastery-approach goals perform better in predictive adaptation. It appeared that the adoption of these goals overcame any potential negative effects that were exerted by the other three goals (see motivation and approach goals). This may be because mastery-approach goals are primarily linked to a pattern of adaptive educational outcomes (Sommet and Elliot, 2017). An alternative reason is that students who possess mastery-approach goals have fewer negative psychological responses to wrong social comparisons (Kamarova et al., 2017). This study confirmed that mastery-approach goals are a remarkably powerful goal type (Shih, 2012) that considerably boosts academic adjustment.

### Mediation of Learning Engagement

From the perspective of the variable-centered approach, we found that mastery-approach goals of freshmen increased learning engagement, which was positively related to academic adjustment, and performance-avoidance goals reduced learning engagement, which was negatively associated with academic adjustment. That is, learning engagement mediated the relationship between mastery-approach/performance-avoidance goals and academic adjustment. Therefore, why freshmen with different goal orientations are likely to explain different levels of academic adjustment can be explained by learning engagement. These findings support the integrative development-in-sociocultural-context model (Wang et al., 2019), which assumes that learning engagement is a pathway or process through which personal factors (e.g., motivational beliefs) shape learning outcomes.

In addition to the overall mediation result, we focused on a separate link within the mediation model. On the one hand, our findings support the notion that mastery-approach goals are related to increased academic adjustment and that performance-avoidance goals are the opposite of academic adjustment. Competence beliefs have been established as antecedents of engagement (Wang et al., 2019). This finding is consistent with the mindset theory, which posits that judgments of students regarding their performance have a significant influence on engagement (Weiner, 1985). Students who hold mastery-approach goals are likely to have higher learning engagement, whereas students with performance-avoidance goals will have lower learning engagement. On the other hand, we revealed that learning engagement was positively correlated with academic adjustment, which is consistent with previous studies that showed that learning engagement predicts academic achievement (Wang and Eccles, 2012), academic effort (Strauser et al., 2012), and better adaptation (Reschly and Christenson, 2012). Therefore, learning engagement may be used to assess the adjustment of freshmen in school and identify areas for intervention.

From the person-centered perspective, only approach-oriented goals increased learning engagement, which further increased academic adjustment. That is, learning engagement mediated the link between approach-oriented goals and academic adjustment. Emmons (1989) pointed out that for students, specific goals are important for adapting to the current environment. People with clear achievement goals (approach-oriented) will be courageous enough to make effort, face problems, and adopt positive learning strategies. Our results are in line with Luo et al.’s (2011) study that demonstrated that approach-oriented students have more adaptive results for learning motivation (e.g., self-efficacy), learning engagement, and academic emotions [e.g., test anxiety (Luo et al., 2011; Lo et al., 2017)]. Moreover, students achieving goals facilitate meeting their internal needs, obtaining happiness (Job et al., 2009), and more easily adapting to the learning environment.

### Limitations and Future Directions

Despite the theoretical and practical implications, the limitations of our work should be recognized. Firstly, we only tested freshmen, and thus, our results do not generalize across all students. In the future, studies should further consider the relationships between the dimensions and profiles of achievement goal orientations and academic adjustment in senior college students or primary and middle school students. Secondly, our study was based on a Chinese sample. The levels of achievement goal orientations vary between cultures. Therefore, students’ achievement goal orientations should be compared between collectivism and individualism cultures to determine whether our findings generalize to other cultures. Thirdly, we did not observe a relationship between mastery-avoidance goals and outcomes, and the conclusions for mastery-avoidance and performance-approach goals were inconsistent. The reasons underlying these findings require further exploration, which may include investigations on autonomy and control, achievement motivation, and independent support, which will extend the achievement goal research field and offer future directions for research.

### Implications

Our findings have both theoretical and practical implications. In terms of theoretical significance, we integrated variable-centered (concerning individual changes) and person-centered (concerning individual differences) approaches, which provided an important opportunity to advance the understanding of achievement goal orientations and demonstrated a comprehensive account of the influence factors and underlying mechanisms of academic adjustment. Furthermore, we provided further evidence of how academic adjustment is shaped. The person-centered approach allowed us to reveal different motivation patterns of freshmen and complemented the knowledge gained from traditional variable-centered quantitative methods for studying individual differences.

In regard to the practical implications, our findings highlighted the importance of mastery-approach and approach-oriented goals in promoting adaptive academic adjustment. Given the pivotal role of academic adjustment in student success (van Rooij et al., 2018), improving approach-oriented goals may help to increase academic adjustment in freshmen. It is essential for students to establish approach-oriented goals by accumulating experience by applying learning methods, such as inquiry-based learning, which is associated with goal orientations (Ramnarain and Ramaila, 2016). Moreover, by establishing a mediation model, our findings offer advice for practitioners to understand how achievement goal orientations are linked to academic adjustment. These findings may help in the development of appropriate educational practices. Freshmen who only have approach-oriented goals have greater learning engagement and academic adjustment; therefore, teachers should pay more attention to discovering potential at-risk groups and encourage them to explore their sense of competence to ensure that these students do not neglect their schooling. Furthermore, it is vital for teachers to reduce the emphasis on comparisons between students and minimize the negative impact of competitive environments.

## Conclusion

Based on the 2×2 model of achievement goal orientations (Elliot and McGregor, 2001), we explored the dimensions and profiles of achievement goal orientations that influence academic adjustment and whether learning engagement mediates the relationship between achievement goal orientations and academic adjustment. We concluded that as: (1) Different from hypothesis 1, achievement goal orientations can be divided into four potential profiles: low-motivation, approach-oriented, average, and multiple; (2) learning engagement only mediates the relationship between the two goals (mastery-approach and performance-avoidance goals) and academic adjustment; (3) learning engagement mediates the relationship between approach-oriented goals and academic adjustment. Our findings have theoretical implications for future studies and practical implications for teachers to help develop the goals of students. Our results go beyond the previous studies by providing a depth understanding on how freshmen with differential achievement goal orientations performs academic adjustments. In addition, we highlight the importance role of learning engagement in the relationships between achievement goal orientations and academic adjustment.

## Data Availability Statement

The raw data supporting the conclusions of this article will be made available by the authors, without undue reservation.

## Ethics Statement

The studies involving human participants were reviewed and approved by Academic Ethics Committee of the College of Psychology of Northeast Normal University. Written informed consent from the participants’ legal guardian/next of kin was not required to participate in this study in accordance with the national legislation and the institutional requirements.

## Author Contributions

HW designed the research and acquired the funding. MX wrote the manuscript and recruited the participants. XX analyzed and interpretation of data for the work. YD and WW modified the manuscript. All authors listed have made a substantial, direct, and intellectual contribution to the work, and approved it for publication.

## Funding

This study was funded by the project “Humanities and Social Science Research Project of the Ministry of Education ‘Intervention Research on Adolescents’ Social Emotional Ability from the Perspective of Schools’” (17YJA190012).

## Conflict of Interest

The authors declare that the research was conducted in the absence of any commercial or financial relationships that could be construed as a potential conflict of interest.

## Publisher’s Note

All claims expressed in this article are solely those of the authors and do not necessarily represent those of their affiliated organizations, or those of the publisher, the editors and the reviewers. Any product that may be evaluated in this article, or claim that may be made by its manufacturer, is not guaranteed or endorsed by the publisher.

## References

[ref1] Aderi M. Jdaitawi M. Ishak N. A. Jdaitawi F. (2013). The influence of demographic variables on university students’ adjustment in North Jordan. Int. Educ. Stud. 6, 172–178. doi: 10.5539/ies.v6n2p172

[ref2] Arjanggi R. Kusumaningsih L. P. S. (2016). The correlation between social anxiety and academic adjustment among freshmen. Procedia Soc. Behav. Sci. 219, 104–107. doi: 10.1016/j.sbspro.2016.04.049

[ref3] Auvinen T. Hakulinen L. Malmi L. (2015). Increasing students’ awareness of their behavior in online learning environments with visualizations and achievement badges. IEEE Trans. Learn. Technol. 8, 261–273. doi: 10.1109/tlt.2015.2441718

[ref4] Awad A. M. AlAmodi A. A. Shareef M. A. Alsheikh A. J. Mahmod A. I. Daghistany A. O.. (2014). The summer premedical program for matriculating medical students: a student-led initiative. Adv. Physiol. Educ. 38, 56–61. doi: 10.1152/advan.00085.2013, PMID: 24585471

[ref5] Baker R. W. Siryk B. (1984). Measuring adjustment to college. J. Couns. Psychol. 31, 179–189. doi: 10.1037/0022-0167.31.2.179

[ref6] Baranik L. E. Stanley L. J. Bynum B. H. Lance C. E. (2010). Examining the construct validity of mastery-avoidance achievement goals: a meta-analysis. Hum. Perform. 23, 265–282. doi: 10.1080/08959285.2010.488463

[ref7] Barashev A. Li G. (2017). “Personal trait predicting work engagement in crowdsourcing through achievement goals: mediation analyses.” in *Proceedings of the 8th International Conference on E-business, Management and Economics*, 28–32.

[ref8] Bergman L. R. Trost K. (2006). The person-oriented versus the variable-oriented approach: are they complementary, opposites, or exploring different worlds? Merrill Palmer Q. 52, 601–632. doi: 10.1353/mpq.2006.0023

[ref9] Bipp T. Kleingeld A. Schelp L. (2021). Achievement goals and goal progress as drivers of work engagement. Psychol. Rep. 124, 2180–2202. doi: 10.1177/0033294120959778, PMID: 32967531

[ref10] Butler R. (2014). “Motivation in educational contexts: Does gender matter?,” in Advances in child development and behavior, Vol. 47: The role of gender in educational contexts and outcomes. eds. Liben L. S. Bigler R. S. (San Diego: Elsevier Academic Press), 1–41. 10.1016/bs.acdb.2014.05.001 25344992

[ref11] Cheng R. W. Y. Lam S. F. (2013). The interaction between social goals and self-construal on achievement motivation. Contemp. Educ. Psychol. 38, 136–148. doi: 10.1016/j.cedpsych.2013.01.001

[ref12] Crede M. Niehorster S. (2012). Adjustment to college as measured by the Student Adaptation to College Questionnaire: a quantitative review of its structure and relationships with correlates and consequences. Educ. Psychol. Rev. 24, 133–165. doi: 10.1007/s10648-011-9184-5

[ref14] Daniels L. M. Haynes T. L. Stupnisky R. H. Perry R. P. Newall N. E. Pekrun R. (2008). Individual differences in achievement goals: a longitudinal study of cognitive, emotional, and achievement outcomes. Contemp. Educ. Psychol. 33, 584–608. doi: 10.1016/j.cedpsych.2007.08.002

[ref15] Darlow V. Norvilitis J. M. Schuetze P. (2017). The relationship between helicopter parenting and adjustment to college. J. Child Fam. Stud. 26, 2291–2298. doi: 10.1007/s10826-017-0751-3

[ref16] Datu J. A. D. (2018). Flourishing is associated with higher academic achievement and engagement in Filipino undergraduate and high school students. J. Happiness Stud. 19, 27–39. doi: 10.1007/s10902-016-9805-2

[ref17] Daumiller M. Rinas R. Olden D. Dresel M. (2021). Academics’ motivations in professional training courses: effects on learning engagement and learning gains. Int. J. Acad. Dev. 26, 7–23. doi: 10.1080/1360144X.2020.1768396

[ref18] de Lange A. H. Van Yperen N. W. Van der Heijden B. I. J. M. Bal P. M. (2010). Dominant achievement goals of older workers and their relationship with motivation-related outcomes. J. Vocat. Behav. 77, 118–125. doi: 10.1016/j.jvb.2010.02.013

[ref19] Dweck C. S. (1986). Motivational processes affecting learning. Am. Psychol. 41, 1040–1048. doi: 10.1037/0003-066X.41.10.1040

[ref20] Elliot A. J. (1999). Approach and avoidance motivation and achievement goals. Educ. Psychol. 34, 169–189. doi: 10.1207/s15326985ep3403_3

[ref21] Elliot A. J. Church M. A. (1997). A hierarchical model of approach and avoidance achievement motivation. J. Pers. Soc. Psychol. 72, 218–232. doi: 10.1037/0022-3514.72.1.218 10234849

[ref22] Elliot A. J. Harackiewicz J. M. (1996). Approach and avoidance achievement goals and intrinsic motivation: a mediational analysis. J. Pers. Soc. Psychol. 70, 461–475. doi: 10.1037/0022-3514.70.3.461 8014838

[ref23] Elliot A. J. McGregor H. A. (2001). A 2 × 2 achievement goal framework. J. Pers. Soc. Psychol. 80, 501–519. doi: 10.1037/0022-3514.80.3.501, PMID: 11300582

[ref24] Elliot A. J. Murayama K. (2008). On the measurement of achievement goals: critique, illustration, and application. J. Educ. Psychol. 100, 613–628. doi: 10.1037/0022-0663.100.3.613

[ref25] Emmons R. A. (1989). “The personal striving approach to personality” in Goal Concepts in Personality and Social Psychology ed. Pervin L. A. (Hillsdale, NJ: Erlbaum), 87–126.

[ref26] Fang L. T. Shi K. Zhang F. H. (2008). Research on reliability and validity of Utrecht Work Engagement Scale-Student. Chin. J. Clin. Psychol. 16, 618–620.

[ref27] Feng T. Y. Su T. Hu X. W. Li H. (2006). The development of a test about learning adjustment of undergraduate. Acta Psychol. Sin. 5, 762–769.

[ref28] García-Ros R. Pérez-González F. Tomás J. M. Fernández I. (2018). The Schoolwork Engagement Inventory: factorial structure, measurement invariance by gender and educational level, and convergent validity in secondary education (12–18 years). J. Psychoeduc. Assess. 36, 588–603. doi: 10.1177/0734282916689235

[ref29] Gardiner G. Lee D. Baranski E. Funder D. International Situations Project (2020). Happiness around the world: a combined etic-emic approach across 63 countries. PLoS One 15:0242718. doi: 10.1371/journal.pone.0242718, PMID: 33296388 PMC7725360

[ref30] Gillet N. Lafreniere M. A. K. Huyghebaert T. Fouquereau E. (2015). Autonomous and controlled reasons underlying achievement goals: implications for the 3 × 2 achievement goal model in educational and work settings. Motiv. Emot. 39, 858–875. doi: 10.1007/s11031-015-9505-y

[ref31] Gonçalves T. Niemivirta M. Lemos M. S. (2017). Identification of students’ multiple achievement and social goal profiles and analysis of their stability and adaptability. Learn. Individ. Differ. 54, 149–159. doi: 10.1016/j.lindif.2017.01.019

[ref32] Hayes A. F. (2018). Partial, conditional, and moderated moderated mediation: quantification, inference, and interpretation. Commun. Monogr. 85, 4–40. doi: 10.1080/03637751.2017.1352100

[ref33] Hayes A. F. Scharkow M. (2013). The relative trustworthiness of inferential tests of the indirect effect in statistical mediation analysis: does method really matter? Psychol. Sci. 24, 1918–1927. doi: 10.1177/0956797613480187, PMID: 23955356

[ref34] Honicke T. Broadbent J. Fuller-Tyszkiewicz M. (2020). Learner self-efficacy, goal orientation, and academic achievement: exploring mediating and moderating relationships. High. Educ. Res. Dev. 39, 689–703. doi: 10.1080/07294360.2019.1685941

[ref35] Howell A. J. Watson D. C. (2007). Procrastination: associations with achievement goal orientation and learning strategies. Pers. Individ. Differ. 43, 167–178. doi: 10.1016/j.paid.2006.11.017

[ref36] Iacobucci D. (2012). Mediation analysis and categorical variables: the final frontier. J. Consum. Psychol. 22, 582–594. doi: 10.1016/j.jcps.2012.03.006 PMC3501728 23180961

[ref37] Jansen In de Wal J. Hornstra L. Prins F. J. Peetsma T. van der Veen I. (2016). The prevalence, development and domain specificity of elementary school students’ achievement goal profiles. Educ. Psychol. 36, 1300–1319. doi: 10.1080/01443410.2015.1035698

[ref38] Job V. Langens T. A. Brandstatter V. (2009). Effects of achievement goal striving on well-being: the moderating role of the explicit achievement motive. Personal. Soc. Psychol. Bull. 35, 983–996. doi: 10.1177/0146167209336606, PMID: 19498069

[ref39] Kamarova S. Chatzisarantis N. L. D. Hagger M. S. Lintunen T. Hassandra M. Papaioannou A. (2017). Effects of achievement goals on perceptions of competence in conditions of unfavourable social comparisons: the mastery goal advantage effect. Br. J. Educ. Psychol. 87, 630–646. doi: 10.1111/bjep.12168, PMID: 28603871

[ref40] Kaplan A. Maehr M. L. (2007). The contributions and prospects of goal orientation theory. Educ. Psychol. Rev. 19, 141–184. doi: 10.1007/s10648-006-9012-5

[ref41] Knief U. Forstmeier W. (2018). Violating the normality assumption may be the lesser of two evils. bioRxiv. doi: 10.1101/498931, [Epub ahead of print] PMC8613103 33963496

[ref42] Lanza S. T. Cooper B. R. (2016). Latent class analysis for developmental research. Child Dev. Perspect. 10, 59–64. doi: 10.1111/cdep.12163, PMID: 31844424 PMC6914261

[ref43] León S. P. Augusto-Landa J. M. García-Martínez I. (2021). Moderating factors in university students’ self-evaluation for sustainability. Sustainability 13:4199. doi: 10.3390/su13084199

[ref44] León S. P. García-Martínez I. (2021). Impact of the provision of PowerPoint slides on learning. Comput. Educ. 173:104283. doi: 10.1016/j.compedu.2021.104283

[ref45] Li Y. Lynch A. D. Kalvin C. Liu J. Lerner R. M. (2011). Peer relationships as a context for the development of school engagement during early adolescence. Int. J. Behav. Dev. 35, 329–342. doi: 10.1177/0165025411402578

[ref46] Li Y. Yao C. Zeng S. Wang X. Lu T. Li C.. (2019). How social networking site addiction drives university students’ academic achievement: the mediating role of learning engagement. J. Pac. Rim Psychol. 13:19. doi: 10.1017/prp.2019.12

[ref47] Lin Y. G. McKeachie W. J. Kim Y. C. (2003). College student intrinsic and/or extrinsic motivation and learning. Learn. Individ. Differ. 13, 251–258. doi: 10.1016/S1041-6080(02)00092-4

[ref48] Lindell A. K. Killoren S. E. Campione-Barr N. (2020). Parent-child relationship quality and emotional adjustment among college students: the role of parental financial support. J. Soc. Pers. Relat. doi: 10.1177/0265407520964870, [Epub ahead of print]

[ref49] Linnenbrink-Garcia L. Wormington S. V. (2019). “An integrative perspective for studying motivation in relation to engagement and learning,” in The Cambridge Handbook of Motivation and Learning eds. Renninger K. A. Hidi S. E. (Cambridge: Cambridge University Press), 739–758.

[ref50] Liu X. S. (2013). Statistical Power Analysis for the Social and Behavioral Sciences: Basic and Advanced Techniques. 1st *Edn*. New York, NY: Routledge.

[ref51] Liu H. J. Guo D. J. (2003). A research of the relationship between pretest anxiety, achievement goal orientation and test performance. Psychol. Dev. Educ. 2, 64–68.

[ref52] Liu H. Yao M. Li J. (2020). Chinese adolescents’ achievement goal profiles and their relation to academic burnout, learning engagement, and test anxiety. Learn. Individ. Differ. 83–84:101945. doi: 10.1016/j.lindif.2020.101945

[ref53] Lo M. T. Chen S. K. Lin S. S. J. (2017). Groups holding multiple achievement goals in the math classroom: profile stability and cognitive and affective outcomes. Learn. Individ. Differ. 57, 65–76. doi: 10.1016/j.lindif.2017.06.001

[ref54] Luo W. Paris S. G. Hogan D. Luo Z. (2011). Do performance goals promote learning? A pattern analysis of Singapore students’ achievement goals. Contemp. Educ. Psychol. 36, 165–176. doi: 10.1016/j.cedpsych.2011.02.003

[ref55] Meece J. L. Anderman E. M. Anderman L. H. (2006). Classroom goal structure, student motivation, and academic achievement. Annu. Rev. Psychol. 57, 487–503. doi: 10.1146/annurev.psych.56.091103.070258, PMID: 16318604

[ref56] Michou A. Matos L. Gargurevich R. Gumus B. Herrera D. (2016). Building on the enriched hierarchical model of achievement motivation: autonomous and controlling reasons underlying mastery goals. Psychol. Belg. 56, 269–287. doi: 10.5334/pb.281, PMID: 30479440 PMC5854211

[ref57] Montgomery S. Gregg D. H. Somers C. L. Pernice-Duca F. Hoffman A. Beeghly M. (2019). Intrapersonal variables associated with academic adjustment in United States college students. Curr. Psychol. 38, 40–49. doi: 10.1007/s12144-016-9533-0

[ref58] Morrison G. M. Cosden M. A. (1997). Risk, resilience, and adjustment of individuals with learning disabilities. Learn. Disabil. Q. 20, 43–60. doi: 10.2307/1511092

[ref59] Mouratidis A. Michou A. Demircioglu A. N. Sayil M. (2017). Different goals, different pathways to success: performance-approach goals as direct and mastery-approach goals as indirect predictors of grades in mathematics. Learn. Individ. Differ. 61, 127–135. doi: 10.1016/j.lindif.2017.11.017

[ref60] Mouratidis A. Vansteenkiste M. Michou A. Lens W. (2013). Perceived structure and achievement goals as predictors of students’ self-regulated learning and affect and the mediating role of competence need satisfaction. Learn. Individ. Differ. 23, 179–186. doi: 10.1016/j.lindif.2012.09.001

[ref61] Muthén L. K. Muthén B. O. (1998–2015). Mplus User’s Guide. 8th *Edn*. Los Angeles, CA: Muthén & Muthén.

[ref62] Nadon L. Babenko O. Chazan D. Daniels L. M. (2020). Burning out before they start? An achievement goal theory perspective on medical and education students. Soc. Psychol. Educ. 23, 1055–1071. doi: 10.1007/s11218-020-09572-0

[ref63] Nerstad C. G. L. Caniels M. C. J. Roberts G. C. Richardsen A. M. (2020). Perceived motivational climates and employee energy: the mediating role of basic psychological needs. Front. Psychol. 11:1509. doi: 10.3389/fpsyg.2020.01509, PMID: 32754087 PMC7365864

[ref64] Niemivirta M. (2002). “Individual differences and developmental trends in motivation: integrating person-centered and variable-centered methods,” in Advances in Motivation and Achievement, Vol. 12. eds. Pintrich P. R. Maehr M. L. (Amsterdam: JAI Press), 241–275.

[ref65] Nylund K. L. (2007). Latent transition analysis: modeling extensions and an application to peer victimization. doctoral dissertation. Los Angeles, CA: University of California.

[ref66] Peixoto F. Monteiro V. Mata L. Sanches C. Pipa J. Almeida L. S. (2016). “To be or not to be retained … that’s the question!” Retention, self-esteem, self-concept, achievement goals, and grades. Front. Psychol. 7:1550. doi: 10.3389/fpsyg.2016.01550, PMID: 27790167 PMC5062915

[ref67] Pek J. Wong O. Wong A. (2018). How to address non-normality: a taxonomy of approaches, reviewed, and illustrated. Front. Psychol. 9:2104. doi: 10.3389/fpsyg.2018.02104, PMID: 30459683 PMC6232275

[ref68] Pekrun R. Elliot A. J. Maier M. A. (2006). Achievement goals and discrete achievement emotions: a theoretical model and prospective test. J. Educ. Psychol. 98, 583–597. doi: 10.1037/0022-0663.98.3.583

[ref69] Phan H. P. (2016). Interrelations that foster learning: an investigation of two correlational studies. Int. J. Psychol. 51, 185–195. doi: 10.1002/ijop.12127, PMID: 25501749

[ref70] Poortvliet P. M. Anseel F. Theuwis F. (2015). Mastery-approach and mastery-avoidance goals and their relation with exhaustion and engagement at work: the roles of emotional and instrumental support. Work Stress. 29, 150–170. doi: 10.1080/02678373.2015.1031856

[ref71] Poortvliet P. M. Perdeck J. (2014). Mastery-approach goals and self-efficacy as predictors of burnout and work engagement: the adaptive role of team-member exchange quality. Gedrag Organisatie 27, 188–212.

[ref72] Ramnarain U. D. Ramaila S. (2016). The achievement goal orientations of South African first year university physics students. Int. J. Sci. Math. Educ. 14, S81–S105. doi: 10.1007/s10763-014-9590-5

[ref73] Reschly A. L. Christenson S. L. (2012). “Jingle, jangle, and conceptual haziness: evolution and future directions of the engagement construct,” in Handbook of Research on Student Engagement. eds. Christenson S. L. Reschly A. L. Wylie C. (New York, NY: Springer), 3–19.

[ref74] Ryan A. M. Shim S. S. (2006). Social achievement goals: the nature and consequences of different orientations toward social competence. Pers. Soc. Psychol. Bull. 32, 1246–1263. doi: 10.1177/0146167206289345, PMID: 16902243

[ref75] Ryan A. M. Shim S. S. (2008). An exploration of young adolescents’ social achievement goals and social adjustment in middle school. J. Educ. Psychol. 100, 672–687. doi: 10.1037/0022-0663.100.3.672

[ref76] Salmela-Aro K. Upadaya K. (2012). The schoolwork engagement inventory: Energy, Dedication, and Absorption (EDA). Eur. J. Psychol. Assess. 28, 60–67. doi: 10.1027/1015-5759/a000091 PMC7930215 33679565

[ref77] Schaufeli W. B. Martínez I. M. Pinto A. M. Salanova M. Barker A. B. (2002). Burnout and engagement in university students a cross-national study. J. Cross-Cult. Psychol. 33, 464–481. doi: 10.1177/0022022102033005003

[ref78] Senko C. Freund A. M. (2015). Are mastery-avoidance achievement goals always detrimental? An adult development perspective. Motiv. Emot. 39, 477–488. doi: 10.1007/s11031-015-9474-1

[ref79] Shih S. S. (2008). The relation of self-determination and achievement goals to Taiwanese eighth graders’ behavioral and emotional engagement in schoolwork. Elem. School J. 108, 313–334. doi: 10.1086/528974

[ref80] Shih S. S. (2012). An examination of academic burnout versus work engagement among Taiwanese adolescents. J. Educ. Res. 105, 286–298. doi: 10.1080/00220671.2011.629695

[ref81] Skinner E. A. Pitzer J. R. (2012). “Developmental dynamics of student engagement, coping, and everyday resilience,” in Handbook of Research on Student Engagement. eds. Christenson S. L. Reschly A. L. Wylie C. (New York, NY: Springer), 21–44.

[ref82] Skinner E. A. Pitzer J. R. Steele J. S. (2016). Can student engagement serve as a motivational resource for academic coping, persistence, and learning during late elementary and early middle school? Dev. Psychol. 52, 2099–2117. doi: 10.1037/dev0000232, PMID: 27893248

[ref83] Sommet N. Elliot A. J. (2017). Achievement goals, reasons for goal pursuit, and achievement goal complexes as predictors of beneficial outcomes: is the influence of goals reducible to reasons? J. Educ. Psychol. 109, 1141–1162. doi: 10.1037/edu0000199

[ref84] Stern M. Hertel S. (2020). Profiles of parents’ beliefs about their child’s intelligence and self-regulation: a latent profile analysis. Front. Psychol. 11:610262. doi: 10.3389/fpsyg.2020.610262, PMID: 33362670 PMC7756061

[ref85] Strauser D. R. O’Sullivan D. Wong A. W. K. (2012). Work personality, work engagement, and academic effort in a group of college students. J. Employ. Couns. 49, 50–61. doi: 10.1002/j.2161-1920.2012.00006.x

[ref86] Tapola A. Jaakkola T. Niemivirta M. (2014). The influence of achievement goal orientations and task concreteness on situational interest. J. Exp. Bot. 82, 455–479. doi: 10.1080/00220973.2013.813370

[ref87] Tapola A. Niemivirta M. (2008). The role of achievement goal orientations in students’ perceptions of and preferences for classroom environment. Br. J. Educ. Psychol. 78, 291–312. doi: 10.1348/000709907X205272, PMID: 17535519

[ref89] Tian L. L. Yu T. T. Huebner E. S. (2017). Achievement goal orientations and adolescents’ subjective well-being in school: the mediating roles of academic social comparison directions. Front. Psychol. 8:37. doi: 10.3389/fpsyg.2017.00037, PMID: 28197109 PMC5281619

[ref90] Tuominen H. Niemivirta M. Lonka K. Salmela-Aro K. (2020). Motivation across a transition: changes in achievement goal orientations and academic well-being from elementary to secondary school. Learn. Individ. Differ. 79:101854. doi: 10.1016/j.lindif.2020.101854

[ref91] Tuominen-Soini H. Salmela-Aro K. Niemivirta M. (2008). Achievement goal orientations and subjective well-being: a person-centred analysis. Learn. Instr. 18, 251–266. doi: 10.1016/j.learninstruc.2007.05.003

[ref92] Tuominen-Soini H. Salmela-Aro K. Niemivirta M. (2011). Stability and change in achievement goal orientations: a person-centered approach. Contemp. Educ. Psychol. 36, 82–100. doi: 10.1016/j.cedpsych.2010.08.002

[ref93] Tuominen-Soini H. Salmela-Aro K. Niemivirta M. (2012). Achievement goal orientations and academic well-being across the transition to upper secondary education. Learn. Individ. Differ. 22, 290–305. doi: 10.1016/j.lindif.2012.01.002

[ref94] Upadyaya K. Salmela-Aro K. (2013). Development of school engagement in association with academic success and well-being in varying social contexts: a review of empirical research. Eur. Psychol. 18, 136–147. doi: 10.1027/1016-9040/a000143

[ref95] van Dam A. Noordzij G. Born M. (2020). Social workers and recovery from stress. J. Soc. Work. 21, 999–1018. doi: 10.1177/1468017320911350

[ref96] van Rooij E. C. M. Jansen E. P. W. A. van de Grift W. J. C. M. (2018). First-year university students’ academic success: the importance of academic adjustment. Eur. J. Psychol. Educ. 33, 749–767. doi: 10.1007/s10212-017-0347-8

[ref97] Van Yperen N. W. Blaga M. Postmes T. (2014). A meta-analysis of self-reported achievement goals and nonself-report performance across three achievement domains (work, sports, and education). PLoS One 9:e93594. doi: 10.1371/journal.pone.0093594, PMID: 24699695 PMC3974764

[ref98] Van Yperen N. W. Blaga M. Postmes T. (2015). A meta-analysis of the impact of situationally induced achievement goals on task performance. Hum. Perform. 28, 165–182. doi: 10.1080/08959285.2015.1006772

[ref99] Van Yperen N. W. Elliot A. J. Anseel F. (2009). The influence of mastery-avoidance goals on performance improvement. Eur. J. Soc. Psychol. 39, 932–943. doi: 10.1002/ejsp.590

[ref100] Vansteenkiste M. Smeets S. Soenens B. Lens W. Matos L. Deci E. L. (2010). Autonomous and controlled regulation of performance-approach goals: their relations to perfectionism and educational outcomes. Motiv. Emot. 34, 333–353. doi: 10.1007/s11031-010-9188-3

[ref101] Vizoso C. Rodriguez C. Arias-Gundin O. (2018). Coping, academic engagement and performance in university students. High. Educ. Res. Dev. 37, 1515–1529. doi: 10.1080/07294360.2018.1504006

[ref102] Wang M.-T. Degol J. L. Henry D. A. (2019). An integrative development-in-sociocultural-context model for children’s engagement in learning. Am. Psychol. 74, 1086–1102. doi: 10.1037/amp0000522, PMID: 31829690

[ref103] Wang M. T. Eccles J. S. (2012). Social support matters: longitudinal effects of social support on three dimensions of school engagement from middle to high school. Child Dev. 83, 877–895. doi: 10.1111/j.1467-8624.2012.01745.x, PMID: 22506836

[ref104] Wang M.-T. Eccles J. S. (2013). School context, achievement motivation, and academic engagement: a longitudinal study of school engagement using a multidimensional perspective. Learn. Instr. 28, 12–23. doi: 10.1016/j.learninstruc.2013.04.002

[ref105] Warren M. T. Schonert-Reichl K. A. Gill R. Gadermann A. M. Oberle E. (2021). Naturalistic development of trait mindfulness: a longitudinal examination of victimization and supportive relationships in early adolescence. PLoS One 16:e0250960. doi: 10.1371/journal.pone.0250960, PMID: 33961643 PMC8104379

[ref106] Weiner B. (1985). An attributional theory of achievement motivation and emotion. Psychol. Rev. 92, 548–573. doi: 10.1037/0033-295X.92.4.548, PMID: 3903815

[ref107] Wigfield A. Cambria J. (2010). Students’ achievement values, goal orientations, and interest: definitions, development, and relations to achievement outcomes. Dev. Rev. 30, 1–35. doi: 10.1016/j.dr.2009.12.001

[ref108] Wormington S. V. Linnenbrink-Garcia L. (2017). A new look at multiple goal pursuit: the promise of a person-centered approach. Educ. Psychol. Rev. 29, 407–445. doi: 10.1007/s10648-016-9358-2

[ref109] Xiang S. Y. Liu Y. Bai L. (2017). Parenting styles and adolescents’ school adjustment: investigating the mediating role of achievement goals within the 2 × 2 framework. Front. Psychol. 8:1809. doi: 10.3389/fpsyg.2017.01809, PMID: 29085321 PMC5650634

[ref110] Xu X. Xu G. Liu M. Deng C. (2020). Influence of parental academic involvement on the achievement goal orientations of high school students in China: a latent growth model study. Br. J. Educ. Psychol. 90, 700–718. doi: 10.1111/bjep.12326, PMID: 31680248

[ref111] Yu J. McLellan R. (2019). Beyond academic achievement goals: the importance of social achievement goals in explaining gender differences in self-handicapping. Learn. Individ. Differ. 69, 33–44. doi: 10.1016/j.lindif.2018.11.010

[ref112] Zhang Y. Watermann R. Daniel A. (2016). Are multiple goals in elementary students beneficial for their school achievement? A latent class analysis. Learn. Individ. Differ. 51, 100–110. doi: 10.1016/j.lindif.2016.08.023

